# The Roles of Whole-Genome and Small-Scale Duplications in the Functional Specialization of *Saccharomyces cerevisiae* Genes

**DOI:** 10.1371/journal.pgen.1003176

**Published:** 2013-01-03

**Authors:** Mario A. Fares, Orla M. Keane, Christina Toft, Lorenzo Carretero-Paulet, Gary W. Jones

**Affiliations:** 1 Integrative and Systems Biology Laboratory, Instituto de Biología Molecular y Celular de Plantas, Consejo Superior de Investigaciones Científicas and Universidad Politécnica de Valencia, Valencia, Spain; 2 Evolutionary Genetics and Bioinformatics Laboratory, Department of Genetics, University of Dublin, Trinity College, Dublin, Ireland; 3 Animal and Bioscience Department, Teagasc, Dunsany, Ireland; 4 Department of Genetics, University of Valencia, Valencia, Spain; 5 Department of Biology, National University of Ireland, Maynooth, Ireland; University of Washington, United States of America

## Abstract

Researchers have long been enthralled with the idea that gene duplication can generate novel functions, crediting this process with great evolutionary importance. Empirical data shows that whole-genome duplications (WGDs) are more likely to be retained than small-scale duplications (SSDs), though their relative contribution to the functional fate of duplicates remains unexplored. Using the map of genetic interactions and the re-sequencing of 27 *Saccharomyces cerevisiae* genomes evolving for 2,200 generations we show that SSD-duplicates lead to neo-functionalization while WGD-duplicates partition ancestral functions. This conclusion is supported by: (a) SSD-duplicates establish more genetic interactions than singletons and WGD-duplicates; (b) SSD-duplicates copies share more interaction-partners than WGD-duplicates copies; (c) WGD-duplicates interaction partners are more functionally related than SSD-duplicates partners; (d) SSD-duplicates gene copies are more functionally divergent from one another, while keeping more overlapping functions, and diverge in their sub-cellular locations more than WGD-duplicates copies; and (e) SSD-duplicates complement their functions to a greater extent than WGD–duplicates. We propose a novel model that uncovers the complexity of evolution after gene duplication.

## Author Summary

Gene duplication involves the doubling of a gene, originating an identical gene copy. Early evolutionary theory predicted that, as one gene copy is performing the ancestral function, the other gene copy, devoid from strong selection constraints, could evolve exploring alternative functions. Because of its potential to generate novel functions, hence biological complexity, gene duplication has been credited with enormous evolutionary importance. The way in which duplicated genes acquire novel functions remains the focus of intense research. Does the mechanism of duplication—duplication of small genome regions versus genome duplication—influence the fate of duplicates? Although it has been shown that the mechanism of duplication determines the persistence of genes in duplicate, a model describing the functional fates of duplicates generated by whole-genome or small-scale duplications remains largely obscure. Here we show that despite the large amount of genetic material originated by whole-genome duplication in the yeast *Saccharomyces cerevisiae*, these duplicates specialized in subsets of ancestral functions. Conversely, small-scale duplicates originated novel functions. We describe and test a model to explain the evolutionary dynamics of duplicates originated by different mechanisms. Our results shed light on the functional fates of duplicates and role of the duplication mechanism in generating functional diversity.

## Introduction

The mechanisms underlying the emergence of novel functions in nature remain a mystery. Gene duplication is believed to be the primary source of new genes and functions and has consequently been credited with great evolutionary importance [1]. Our knowledge on the importance of duplication in functional innovation is impressive, yet our ability to model the functional fate of duplicated genes is highly limited.

A number of studies have attempted to establish a causal link between gene duplication and the emergence of major evolutionary innovations. For example, most Angiosperms have undergone at least one genome duplication (polyploidy) [2], [3] in the Creataceous era, contemporary with the explosion of plant metabolic and physiological diversity [4], [5]. This diversity resulted from the expansion of protein families by gene duplication, including pepsin- and subtilisin-like proteases [6], metacaspases [7], regulatory genes [8] and developmentally important MADS-Box genes [9], [10], [11], [12]. In animals, although much rarer, gene duplications have preceeded the appearence of key developmental features and were concomitant with major events of species diversification [13], [14]. It is tempting to establish a link between gene duplication and biological complexity, but the mechanisms underlying the persistence of genes in duplicate and determining their functional fate remain largely obscure.

Population genetics theory predicts that most duplicated genes return to single copies “shortly” after duplication because an entirely redundant duplicate will fix deleterious mutations and undergo decay and erosion after duplication [1], [15], [16]. Following this prediction, genes will persist in duplicate in the genome if: (i) gene duplication, hence redundancy, endows organisms with mutational robustness [17]; (ii) there is selection for increased gene dosage [18]; or (iii) gene duplicates have diverged functionally through the partitioning of the ancestral gene functions [19], [20], [21], thereby generating entirely new functions [22], or have diverged in their expression profiles [23]. Because gene dosage is immediately unbalanced after duplication, other factors or mechanisms should counterbalance such a constraint to mediate the persistence of genes in duplicate [24], [25]. These mechanisms remain hitherto a major question in molecular evolution [16], [18], [26].

The mode of gene duplication (WGD or SSD) has been proposed to have a key role in the fate of duplicated genes [27] (although see [28] for the role of species ecology in the functional fate of duplicates), with WGDs being more likely to persist than SSDs, as the former does not upset the stoichiometric balance in the cell [24], [25], [29], [30]. Long-term survival of WGDs in the genome can offer opportunities to generate novel functions, albeit this is constrained by gene dosage balance. Therefore, whether genes and their products resulting from both WGD and SSD are subject to the same evolutionary constraints and have similar potential to generate novel functions is unclear.

Typically, events of functional divergence between duplicated genes can be inferred using evolutionary parameters, assuming that when the protein sequences of duplicates are more divergent so are their functions [31], [32], [33]. However, determining whether two copies of a duplicated gene have identical, similar or different functions requires the concerted and careful examination of the function of each gene product. While this approach is useful at a single gene level, genome-scale analyses of functional divergence between gene duplicates are unfeasible on a gene-by-gene basis. Alternatively, high-throughput methods, such as genetic interactions screening [34], yeast two-hybrid screening [35], [36], [37], [38], [39] and analysis of protein complexes by mass spectrometry [40], [41], [42], [43] provide substantial information that can aid in testing the roles of WGD and SSD in innovation.

Using such high-throughput information, several authors have contributed to the understanding of the role of the modes of gene duplication in the functional divergence of duplicated genes. For example, Wagner analyzed the number of shared interactions between duplicated genes in a network as a crude measure of their functional overlap [44]. Analysis of various types of networks on a large scale led Conant and Wolfe [45] to the observation of asymmetry and partitioning of genetic interactions (sub-functionalization) between the daughters of genes after WGD in the yeast *S. cerevisiae*. The different contribution of WGD and SSD to functional divergence was also pointed out in another study using information on protein interactions [46]. Finally, Hakes and colleagues [25] used protein interactions and Gene Ontology (GO) annotations as proxies for protein function to show that functional divergence between SSDs is greater than between WGDs, WGDs produce less deleterious effects when deleted and WGDs are usually part of the same protein complexes.

Recently, Costanzo and colleagues [34], [47] have constructed a functional map that includes the genetic interaction profiles (epistasis) for approximately 75% of the genes in *S. cerevisiae*. Two genes are considered to interact when the phenotypic effect of a variant of one gene is aggravated (synergistic or negative epistasis) or alleviated (antagonistic or positive epistasis) by variation in the second gene [48]. In the extreme, these combinations can lead to synthetic lethality in which mutation of a single gene, although having little or no effect on the cell in isolation, results in cell death when combined with a mutation in a second gene [49], [50]. These interaction profiles provide a means to identify functional relationships between duplicated genes. Accordingly, VanderSluis and colleagues [51] used genetic interaction profiles to demonstrate that duplicated genes can be functionally redundant, show subtle functional differences, their persistence depends on their dosage and gene copies can show asymmetry in their interaction profiles. Moreover, Jiang and colleagues [52] unearthed the role of gene duplication in the evolution of genetic interaction networks and in mediating functional diversification of the interaction partners of a duplicate.

Despite their insightful findings, a model that describes the contribution of the mode of gene duplication to innovation is lacking. More precisely, the different propensities of WGDs and SSDs to generate novel functions that depart from the ancestral ones remain to be inferred.

We used the genetic interaction dataset of Costanzo and colleagues and a large-scale evolution experiment across which we examined mutational dynamics in duplicated genes formed by SSD and WGD. Exhaustive analysis of interaction profiles and genome-wide mutational dynamics allowed us to distinguish the role of WGD and SSD in the functional specialization of *S. cerevisiae* genes and shed light on the complexity of the dynamics of evolution by gene duplication. In particular, we show that: (a) SSDs establish more functions and have stronger epistatic effects in the cell than WGDs; (b) SSD is often followed by neo-functionalization while sub-functionalization is likely to follow WGD and (c) we propose and test a model that explain the role of the mechanism of duplication in the functional fate of duplicates.

## Results

### A model for the evolution of functions after gene duplication

**Figure 1:**
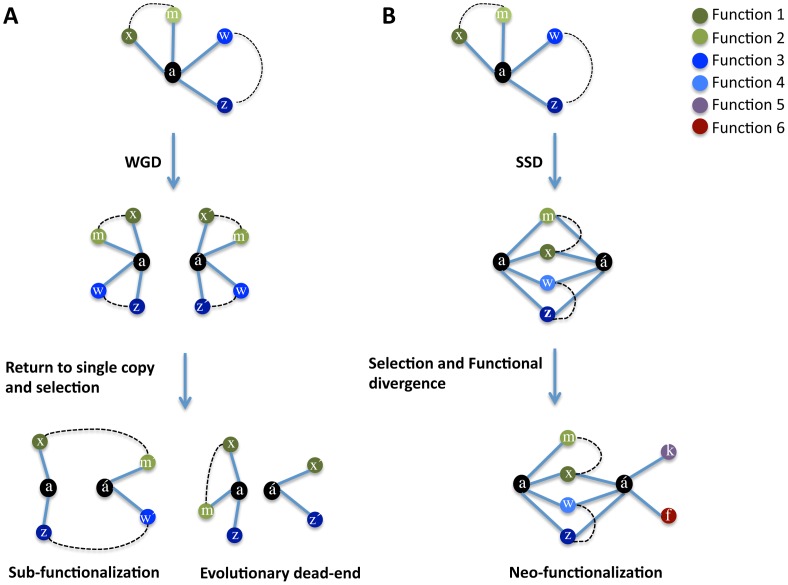
Model of evolution after gene duplication. After whole genome duplication (A), duplicated genes preserve their functions (and therefore genetic interactions: here indicated with colour-labelled circles) interacting (indicated by solid lines between circles) with their partners (x, m, w, z). The partners of a duplicated gene also interact functionally with one another (dashed lines) and are stoichiometrically balanced. Because of genetic redundancy, stochastic loss of genes (functions) takes place, with the final combinations of genes being preserved if they satisfy the overall stoichiometric balance of the cell, with complete partitioning of ancestral functions between the two gene copies (sub-functionalization) being the extreme solution. (B) Duplication of one or few genes in the genome (also known as small-scale duplication: SSD) generates genetic robustness (phenotype resistance to loosing one of the gene copies) if the stoichiometry is not dramatically unbalanced after duplication. This genetic robustness imposes a selective pressure to keep a large overlap in the genetic interaction patterns (functions) of gene copies. The persistence of both gene copies in the genome for long evolutionary periods allows the functional divergence of one gene copy (á) and the acquisition of novel functions (novel genetic interactions: k and f).

Early theory predicts that after gene duplication both copies are functionally redundant and that one of the copies, devoid of selective pressures, degenerates towards non-functionalization in a neutral manner (without consequences for the organism's fitness). We hypothesize that gene duplication immediately re-shapes the fitness landscape of genes and that the shape of the new landscape is dependent upon the mode of duplication. WGD maintains the stoichiometric balance of gene products (Figure 1A) and consequently leads to relaxed selective constraints on both gene copies. These relaxed constraints lead to a stochastic loss of genes so that both copies persist in the genome if the combination of their functional loss does not alter the ancestral function and this combination is not deleterious to the organism (Figure 1A). Conversely, the gene copies formed by SSD persist in the genome if their products do not upset the stoichiometric balance (Figure 1A) or the positive effects on fitness owing to the genetic robustness provided by a second gene copy compensates negative fitness effects of dosage imbalance. The persistence of SSDs facilitates genetic robustness by maintaining overlap in the interaction (function) profiles of the gene copies while generating opportunity for the divergence of one gene copy and the acquisition of novel functions (Figure 1B).

In support of the determinant role of dosage balance in the retention of duplicates and that WGD maintains such a balance is that WGD-duplicated genes have rarely experienced subsequent SSD, they are refractory to copy number variation and WGD-duplicated genes are dosage sensitive, often leading to diseases in humans [29]. The partial or total functional complementation between duplicates reported in several previous studies support the role of genetic robustness in the persistence of duplicates [53], [54], [55], [56], [57].

Our model allows a number of predictions to be made: a) SSDs should complement their function to a greater extent than WGDs; b) SSDs should establish more genetic interactions (GI) than WGDs; c) WGD-duplicated gene copies should partition ancestral functions (sub-functionalize) more readily than SSD-gene copies; d) SSD-gene copies should share more interaction partners and establish more novel interactions (neo-functionalization) than WGD-gene copies and e) the WGDs-interaction partners should be more functionally linked (they should genetically interact between themselves) than those of SSD-interaction partners as the interactions partners for both copies of a WGD should correspond to those of the ancestral pre-duplication gene.

Two key studies present evidence supporting some of the predictions made in this model. The first study is that of Hakes and colleagues [25], who, using protein-protein interaction data and functional similarities, showed that: (a) WGDs exhibit less severe phenotypic effects when deleted than SSDs; (b) WGDs diverged functionally to a lesser extent than SSDs; and (c) WGDs generally encode proteins of the same protein complex. This study however used protein-protein interactions as a proxy for functions, while in this study we focused on genetic interactions. The second study was that of VanderSluis *et al*. [51] which showed that WGDs show stronger negative interactions than SSDs, suggesting greater partitioning of ancestral functions for the former than for the latter.

### SSDs establish more genetic interactions and of stronger effect than WGDs

Previous work, using information contained within protein-protein interactions of *S. cerevisiae*, found that WGDs gene copies show more redundancy, and hence are less essential, than SSDs gene copies [25]. Also, VandersLuis *et al*. [51] examined the difference in the average number of genetic interactions between duplicates, but did not quantify the interactions, which is an important measure of gene redundancy. Here, we examined whether SSDs present more genetic interactions than WGDs and we measured the difference in the strength of interactions between WGDs and SSDs.

We extracted the genetic interaction profiles (762,768 significant interactions with *P*<0.05, according to Supplementary files S4 and S5 from http://drygin.ccbr.utoronto.ca/~costanzo2009/) for 4,464 *S. cerevisiae* genes, which included both singletons and duplicated genes. Of these 4,464, we obtained genetic interaction profiles for 678 duplicated *S. cerevisiae* gene pairs (248 SSDs and 430 WGDs, Table S1 and Table S2 respectively; see Material and Methods). Of the 762,768 significant genetic interactions, 25,003 genetic interactions were established by genes that were in duplicate in the genome (corresponding to the number of genetic interactions once we removed those cases for which the effects of double mutants were not statistically significant when compared to the multiplicative effects of single mutants, *P*>0.05). The number of genetic interactions detected is slightly different from that detected in [51], although is consistent with VanderSluis et al (2010) [51]: albeit we identified marginally less WGDs and marginally more SSDs. The reason for this difference is probably due to the cutoff value used in the BLAST analyses or differences in the methodology used for identity searching (see Material and Methods). Nevertheless, the slight difference between the numbers of genes in both datasets does not affect the conclusions of this study, as on the whole both datasets are very similar. Moreover, we performed the analyses focusing on subsets including 80% of WGDs and SSDs and we were able to reproduce all the conclusions that were obtained in the full datasets (data not shown). The conclusions therefore are very robust to changes in the size of the duplicates datasets.

The functional map of Costanzo and colleagues [47] is based on the synthetic genetic array methodology [58], in which synthetic lethal genetic interactions are systematically mapped by producing single and double mutants [59]. In their study, Costanzo and colleagues [47] identified digenic interactions as those double mutants that show a significant deviation in fitness compared to the multiplicative fitness effects of the two single mutants, that is, epistasis (hereafter referred to as ε, with ε^−^ referring to negative epistasis and ε^+^ to positive epistasis) [60]. Defects were measured in terms of colony size.

**Figure 2:**
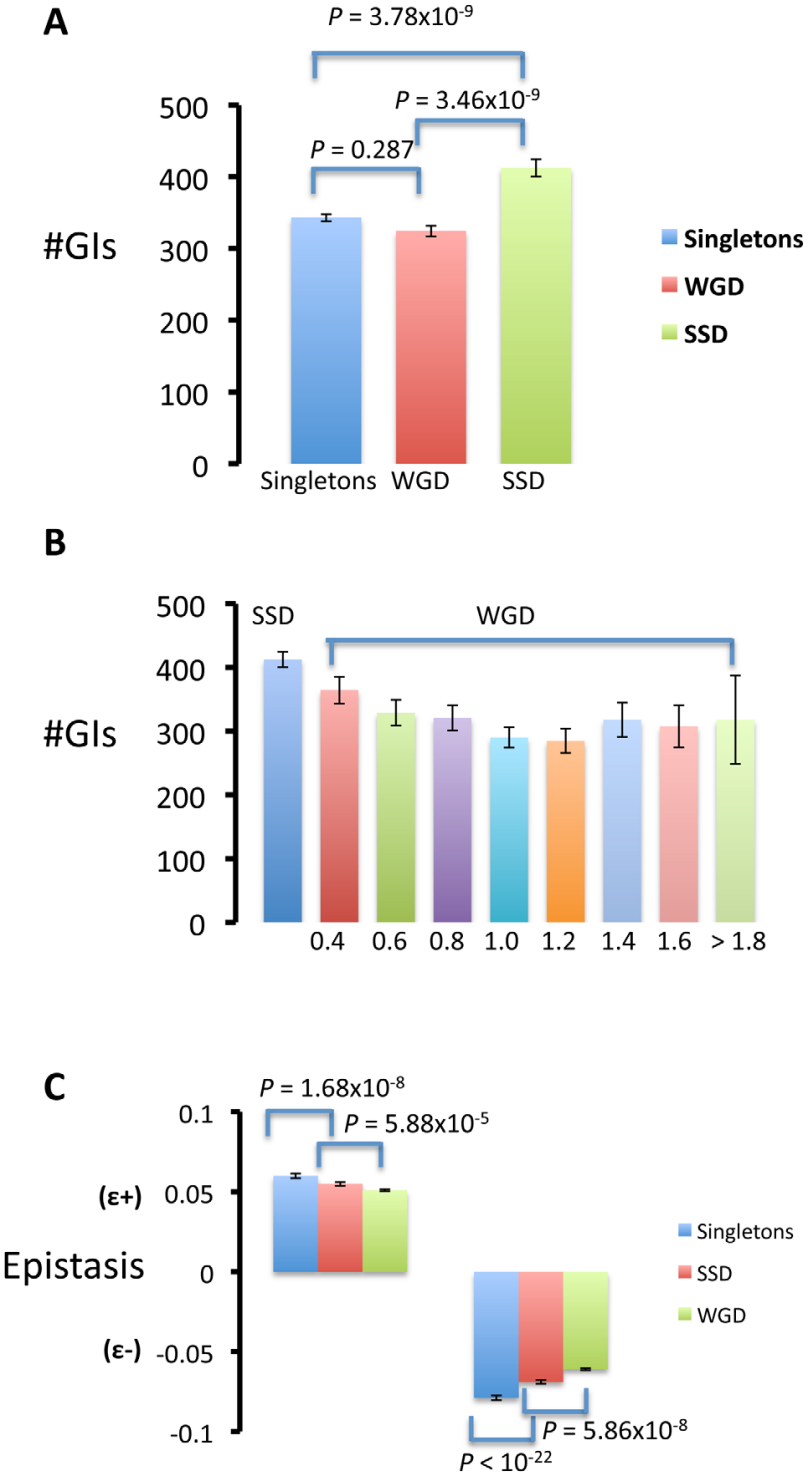
Gene duplicated by small-scale duplications (SSD) present a larger number of genetic interactions (#GI) than those duplicated by whole-genome duplication (WGD) and singletons. (A) Gene duplicated by small-scale duplications (SSD) present a larger number of genetic interactions (#GI) than those duplicated by whole-genome duplication (WGD) and singletons. (B) To determine whether greater genetic redundancy of WGDs may spuriously generate lower number of genetic interactions, we generated bins of duplicated genes according to the JTT amino acid divergences between the gene copies. These bins ranged between 0.4 (that included duplicated genes with the two copies diverging up to 0.4 substitutions per site) and 1.8 (when the divergence between the gene copies was equal or greater than 1.8 amino acid substitutions per site). We noticed no significant differences in the number of genetic interactions between the different bins. (C) The epistatic effects (ε), both positive epistasis (ε^+^) and negative epistasis (ε^−^), of singletons were stronger than those of SSD and WGDs, and the effects of SSDs were stronger than those of WGDs. Differences in the number of genetic interactions (#GI) or their effects between the three categories of genes (singletons, SSD and WGD) were identified using Wilcoxon rank test.

Using epistasis data we found that epistatic effects of duplicated genes with other genes in the genome were predominantly synergistic for duplicates formed by both SSD (the mean effect of double mutant: 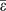 = −0.013) and WGD (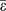 = −0.007), which is in agreement with previous studies [34], [47]. On average, we identified more epistatic interactions for singleton genes (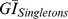 = 342.970) than for WGDs (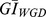 = 324.420) (Figure 2A), suggesting that the copies of WGDs specialized in interacting with a subset of the partners for their ancestral gene (pre-duplication gene). Importantly, we found more genetic interactions for SSDs than for singletons (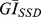 = 412.461) (Figure 2A), suggesting that SSDs have established novel genetic interactions after duplication. Moreover, we identified more epistatic interactions on average for SSDs than for WGDs (*t* = 6.155; *d.f.* = 857,33; *P* = 1,153×10^−9^; Wilcoxon rank test: *P* = 3,465×10^−9^, Figure 2A). These results are consistent with the hypothesis that each gene copy of SSDs preserved on average more ancestral interactions than WGDs and they have established novel interactions once they have specialized in a subset of the ancestral functions (sub-functionalization followed by neo-functionalization, a model previously proposed [22]). However, another possibility is that WGDs may present greater redundancy than SSDs (functional complementation is greater among WGDs) which may buffer the genetic interactions in WGDs, a model proposed in a recent study [51]. To test this possibility, we divided the set of WGDs into bins according to the divergence between the protein sequences of gene copies. According to the buffering model, the mean number of genetic interactions should be lower for bins of low divergence levels (more redundant gene copies) and significantly lower than the mean number of interactions for SSDs. This difference should become significantly diluted at large divergence levels between WGDs. In no bin was this the case and, in fact, the bin with the lowest divergence level for WGDs was the one with the largest number of genetic interactions (Figure 2B). This supports the fact that most duplicated genes in the WGD dataset are no longer redundant after evolving for 100 My [61]. This supports the conclusion that SSDs present more genetic interactions than WGDs and that this is not due to larger redundancy among WGDs.

Because genes with a larger number of interactions should play, on average, a more fundamental role in the cell than those with fewer interactions, we tested whether the number and strength of positive and negative epistasis differred between SSDs and WGDs. Hakes et al. showed, using protein interaction data, that WGDs were more redundant than SSDs as deleting WGDs had less severe effects than deleting SSDs [25] suggesting that WGDs should present less epistasis than SSDs. In agreement with their results, SSDs presented more positive interactions (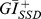 = 181.26) with other genes in the genome than WGDs (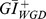 = 152.779; Wilcoxon rank test: *P* = 3.035×10^−5^). Likewise, SSDs presented more negative epistasis (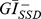 = 231,201) than WGDs (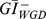 = 171,741) (Wilcoxon rank test: *P* = 0.00034).

We tested whether SSDs duplicates establish stronger epistatic interactions than WGDs, that is, whether deleting a SSD duplicated gene member would have greater effect in combination with other gene deletions than deleting a WGD duplicate. The mean magnitude of positive epistasis for singletons (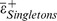 = 0.060) was significantly larger than that for SSDs (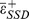 = 0.055) and WGDs (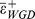 = 0.052) (Figure 2C). The trend was reproduced for negative epistasis: singletons presented stronger average magnitude of epistasis (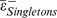 = −0.079) than WGDs (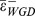 = −0.062) and SSDs (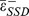 = −0.070). This indicates that the genetic redundancy provided by gene duplication buffers the epistatic effects and points to functional complementation between duplicates. Importantly, SSDs showed stronger epistatic effects than WGDs (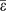 = −0.013, 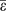 = −0.007; Student t test: *t* = 3.644, *d.f.* = 1058.919, *P* = 2.82×10^−4^), and this trend was also true when examining both positive epistasis (*t* = 4.033, *d.f.* = 1058.803, *P* = 5.889×10^−5^) and negative epistasis (*t* = 5.469, *d.f.* = 892.493, *P* = 5.864×10^−8^). These results suggest that interactions of SSDs are of greater significance for the cell, are more abundant than those of WGDs and point to greater specialization, probably sub-functionalization, of WGDs than SSDs.

### SSD-duplicates gene copies share more genetic interactions than WGD-duplicates

**Figure 3:**
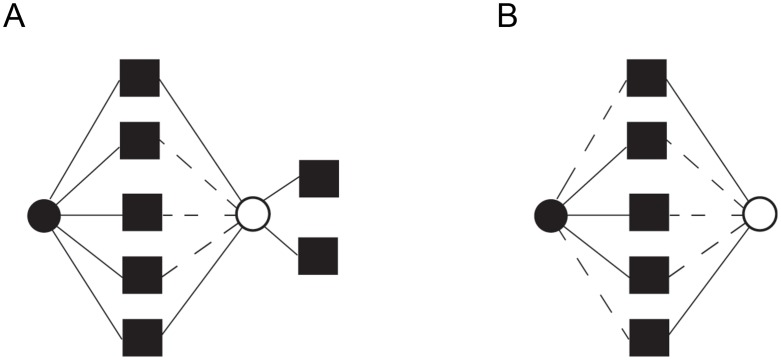
Distinct functional fates for genes duplicated by small-scale duplication (SSD) and whole-genome duplication (WGD). (A) After the duplication of a gene by SSD (circles), one of the gene copies (black circle) maintains the ancestral functions (squares), while the other (white circle) loses (discontinuous lines) some ancestral functions while establishing novel genetic interactions (functions) through the process of neo-functionalization. (B) Genes duplicated by WGD sub-functionalize through the partitioning of ancestral functions so that each gene copy specializes in a subset of the ancestral functions.

The second prediction of our model is that greater genetic redundancy (for example, overlapping functions) in SSDs can allow the generation of novel functions in these duplicated genes. That is, neo-functionalization requires the maintenance of genetic redundancy as a selection pressure to allow the persistence of the gene in duplicate in the genome. Under the model we propose, greater partitioning of ancestral functions among WGDs than SSDs is expected and it is predicted that SSD-gene copies should share more genetic partners than WGD-gene copies (Figure 3). Larger partner sharing among SSDs compared to WGDs may not apply to protein-protein interactions, as shown in Hakes and colleagues [25], especially when duplicated proteins form part of the same complex.

**Figure 4:**
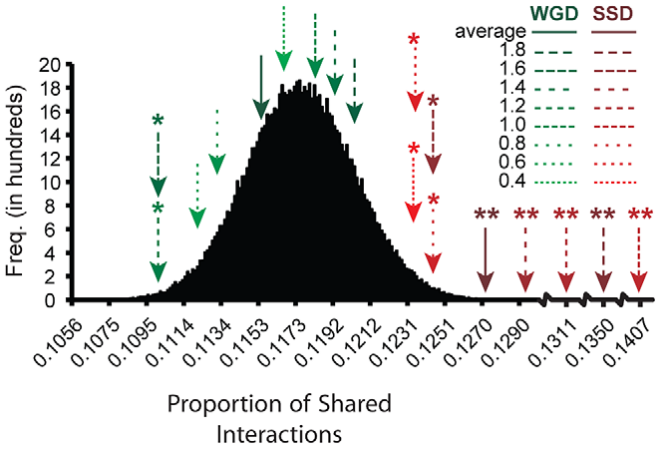
Small-scale duplication (SSD) generates gene copies sharing more ancestral functions than whole-genome duplication (WGD). We tested the partitioning of ancestral functions after duplication by SSD and WGD. We calculated partitioning of ancestral functions by estimating the proportion of shared genetic interactions between the copies of a duplicated gene. This proportion was calculated as *Θ_SSD|WGD_* = (2*n_S(i,j)_*)/(*GI_i_*+*GI_j_*), with *n_S(i,j)_* being the number of genetic interactions (*GI*) in common between gene copies *i* and *j*. To determine the significance of this partitioning (or sharing) we compared *Θ_SSD|WGD_* to that calculated for a distribution of such values estimated from 10^6^ randomly paired singletons. WGDs shared on average (solid green arrow line) as many GIs as random pairs of singletons (for example, the mean indicated by an arrow is within the 90% density of the curve), indicating that they have partitioned their ancestral functions to a point that they could be almost considered as singletons. Conversely, SSD gene copies share ancestral functions (solid red arrow line) significantly more than expected by chance (indicated by asterisks *). The classification of the average number of shared partners between duplicates for different categories of amino acid sequence divergence (amino acid divergence between duplicates was estimated using JTT model) followed the same patterns, with all divergence bins (bins were built with 0.2 divergence levels intervals, except for the first bin) of WGDs (green dashed lines) being not significant while bins of SSDs (red dashed lines) being significant (*: *P*<0.01, **: *P*<10^−6^).

To test this prediction, we examined the divergence in the interaction profiles of the duplicates by estimating the proportion of shared genetic interactions (Θ) between the members (*i* and *j*) of a pair as:
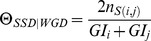
Here *n_S(i,j)_* refers to the number of genetic interactions that are shared between the two copies of a duplicated gene. Using the proportion of shared interactions for duplicates coming from either SSD or WGD, we tested whether generation of novel functions (for example, establishment of novel genetic interactions) was more likely to take place in SSDs while specialization (sub-functionalization: specialization in interacting with a subset of ancestral gene partners) is more likely in WGDs. To do this analysis, we removed from the SSD dataset all those pairs which presented lower sequence divergence than 95% of the WGDs as these were likely to be much younger duplicates and could lead to apparently lower partner sharing in SSDs than in WGDs. The proportion of shared interactions was larger for the members of a SSD duplicate than for those of a WGD duplicate when considering all types of interactions together (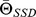 = 0.127, 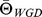 = 0.115, *t* = 2.693, *d.f.* = 514.33, *P* = 0.0073), positive epistatic interactions (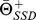 = 0.0622, 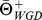 = 0.055, *t* = 3.506, *d.f.* = 573.76, *P* = 0.0124) and negative epistatic interactions (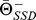 = 0.0708, 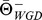 = 0.0616, *t* = 2.810, *d.f.* = 522.11, *P* = 0.0051). Importantly, while SSDs shared significantly more partners than expected from a distribution of shared interactions between randomly paired singletons, this was not the case for WGDs (Figure 4). This pattern was also true for all amino acid sequence divergence levels (Figure 4). Noticeably, when the sequence divergence between both gene copies was high, sharing of partners between them was more apparent, probably due to the lower redundancy (more functional divergence) having less effects on masking true interactions, as proposed by the buffering hypothesis of VanderSluis and colleagues [51]. The same results were obtained for positive and negative epistasis (data not shown). These results further indicate that WGDs have partitioned ancestral functions to the point that each gene copy performs a unique subset of the ancestral functions, while this is not the case for SSDs.

To detail the role of WGD and SSD in sub- and neo-functionalization, respectively, we compared the epistatic interactions between pairs formed by WGD to those originated by SSD taking into account only epistatic interactions between the copies of a duplicated gene. VanderSluis et al. [51] showed stronger epistasis for WGDs than for SSDs. Sub-functionalization would imply strong genetic interactions between the gene copies because both are needed to perform the ancestral, likely essential, function. Neo-functionalization, on the other hand, would require less interactions as one gene copy is almost entirely performing the ancestral function. If this hypothesis were true then we should expect more duplicates to interact epistatically between themselves in the set of WGDs than in the SSD set. In agreement with a previous study [51], WGDs interacted more than SSDs. Copies of a duplicated gene interacted epistatically with each other in 19.8% of WGDs against 12.9% of SSDs, and the difference between these percentages was significant (Fisher exact test: *F* = 1.6638, *P* = 0.0095). Also, sub-functionalization and neo-functionalization after WGD and SSD, respectively, implies that the strength of the interaction should be greater between the gene copies of the set of WGDs than in SSDs. In concert with this prediction, and in addition to the results provided by [51], the epistatic interactions between only the members of a duplication were significantly stronger for WGDs than for SSDs (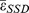 = −0.222, 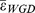 = −0.343; *t* = 2.234, *d.f.* = 89.197, *P* = 0.0279; Wilcoxon rank test: *P* = 0.017). We could not confirm this result for positive and negative epistasis separately due to the lack of statistical power after classifying the types of interactions.

### SSDs diverge functionally more than WGDs

Our model supports neo-functionalization to be more likely in SSDs than WGDs. Comparison of WGDs and SSDs in terms of sequence divergence showed that WGDs diverged less than SSDs in plants [28]. We compared sequence divergence between copies of duplicated genes in the SSD set to that of the WGD set. Neo-functionalization would require dramatic changes in the sequence to perform novel functions while sub-functionalization would subject both gene copies to similar selection pressures as they are required to perform the ancestral function—that is, both gene copies have been co-evolving. It has been previously suggested that both copies of a sub-functionalized duplicate would not be subject to similar selective pressures due to asymmetric partitioning of ancestral functions [22]. However, the difference in the number of functional regions between duplicates that have sub-functionalized is expected to be low.

To test our hypothesis we measured the rate of amino acid divergence between gene copies *i* and *j* (*D_i,j_*), using JTT corrected amino acid distances, for each duplicated gene of the SSD and WGD sets (see material and methods).

As predicted by our hypothesis, the divergence levels between duplicated genes were on average greater for the set of SSDs than for the WGDs set (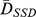 = 0.3082, 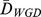 = 0.267, *t* = 2.023, *d.f.* = 580.218, *P* = 0.04). Because sequence divergence is a good indication of functional divergence [31], [32], [33], [62], particularly when divergence is measured between copies of a duplicated gene, the greater divergence between SSDs further suggests that SSDs have a more important role in generating novel functions than WGD and that WGDs co-evolve more than SSDs. This result also predicts that the interaction partners of WGDs should be more functionally related than those of SSDs, as they belonged to the set of interaction partners of a single gene pre-dating the WGD duplication event. This prediction was tested in a previous study by Hakes and colleagues [25] which showed using semantic distances between duplicates that WGDs are more functionally related than SSDs.

To shed more light on the role of SSD and WGD in the functional specialization of duplicated genes, we examined the sub-cellular localization of gene copies formed by both mechanisms of duplication. We extracted information on the cellular localizations of *S. cerevisiae* duplicated genes from the Munich Information Centre for Protein Sequences using the Comprehensive Yeast Genome Database (MIPS *Saccharomyces cerevisiae* genome database: http://mips.helmholtz-muenchen.de/genre/proj/yeast/singleGeneReport.html?entry=yer175c) [63]. We considered two gene copies to present different sub-cellular location if they either had non-overlapping cellular locations or the overlap was not complete. Gene copies localizing to different sub-cellular regions are likely to have developed different functions and vice versa. Different cellular localization of gene copies also buffers the stoichiometric imbalance caused by gene duplication. This hypothesis and our model, predict that SSD-duplicate gene copies will show less overlap in localization than WGD-duplicate gene copies.

As predicted by our model, the number of gene copies resulting from SSD whose duplicate localized to different subcellular locations (330 gene copies corresponding to 165 out of 498 pairs) was significantly larger than that of gene copies resulting from WGD (414; 207 out of 861 duplicates, Table S3) (Fisher exact test: *F* = 2.12, *P* = 7.163×10^−11^). Interestingly, most SSDs overlapped to some degree in their sub-cellular locations, an important finding for the genetic robustness proposed in our model to explain the retention of SSDs.

### WGD-duplicates partners are more functionally linked than SSD-duplicates partners

**Figure 5:**
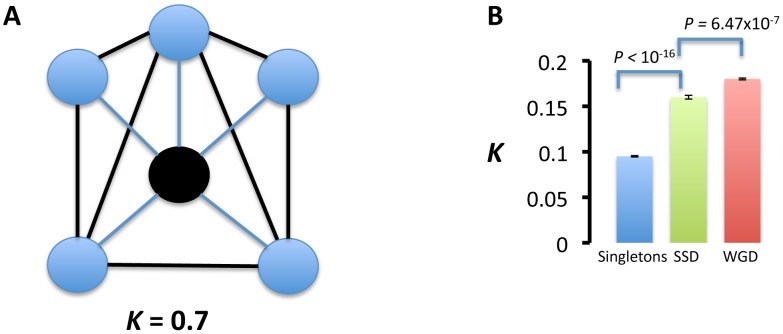
Interaction partners of duplicated genes are more functionally related than those of singletons. (A) We calculated the functional relatedness of the interaction partners (blue circles) of a gene as the proportion of links (*l*) between these partners (black thick lines) taking into account the number of partners (*n*): *k* = 2*l*/*n*(*n*−1). For example, in (A), there are 7 links between the 5 partners of a gene (black circle), which yields k = 2×7/5×4 = 0.7. (B) Clustering coefficients for singletons, small-scale duplications (SSDs) and whole-genome duplications (WGDs). The columns represent the mean clustering coefficient and the standard error of the mean associated to that particular set of genes. Probabilities were calculated by the Wilcoxon rank test. Duplicates interact with genes more functionally related than those with which singletons interact. SSDs interact with genes that are more functionally dispersed (unrelated) than the interaction partners of WGDs.

Another prediction of the proposed model is that SSD-duplicates partners should expand the repertoire of functions more readily than WGD-duplicates partners—that is, SSD-duplicates partners should be less functionally related and hence should interact less than WGD-duplicates partners. To test this prediction, we measured how related were the genes interacting with each copy of a duplicate. Sub-functionalization after duplication would lead to gene copies that interact with highly related functions. Neo-functionalization, on the other hand, would yield gene copies whose partners would be partially unrelated to the ancestral functions (Figure 3). We identified the genetic interactions between the partners of each gene copy. We then measured the clustering between these interaction partners with the assumption in mind that greater clustering involves greater functional relatedeness. We measured the clustering coefficient between the partners of a gene copy as the number of interactions established between these partners (κ):
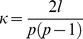
with *l* being the number of links between the partners of a duplicated gene and *p* the number of partners of a duplicated gene. Large clustering coefficients (for example, 0≪κ≤1, Figure 5A) implies that the partners of a duplicated gene are interacting more than expected by chance. We measured this clustering coefficient in two ways. First we estimated κ for singletons and for each gene copy of SSDs and WGDs individually. This yielded larger κ values for WGDs and SSDs than singletons (Figure 5B). WGDs showed significantly larger κ values than SSDs (Figure 5B).

We next joined the set of interaction partners of both copies of a duplicated gene in one group and calculated κ for that group. In agreement with our prediction, κ values were significantly larger for WGDs than for SSDs sets (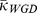 = 0.022, 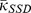 = 0.017, *t* = 12.882, *d.f.* = 646.436, *P*<2.2×10^−16^). These results, in combination with larger functional divergence and partners sharing between SSD-duplicate gene copies than WGD-duplicate gene copies points to larger partitioning of ancestral functions in WGDs than SSDs and greater neo-functionalization in SSDs.

### WGDs are under stronger constraints than SSDs

Our model and results suggest that the functional divergence between members of a WGD is constrained by the need to keep a balanced stoichiometry between duplicates from the same pathway or network, and by their co-evolution. Because of their greater genetic robustness, SSDs should be less constrained to evolve in the short term than WGDs.

**Figure 6:**
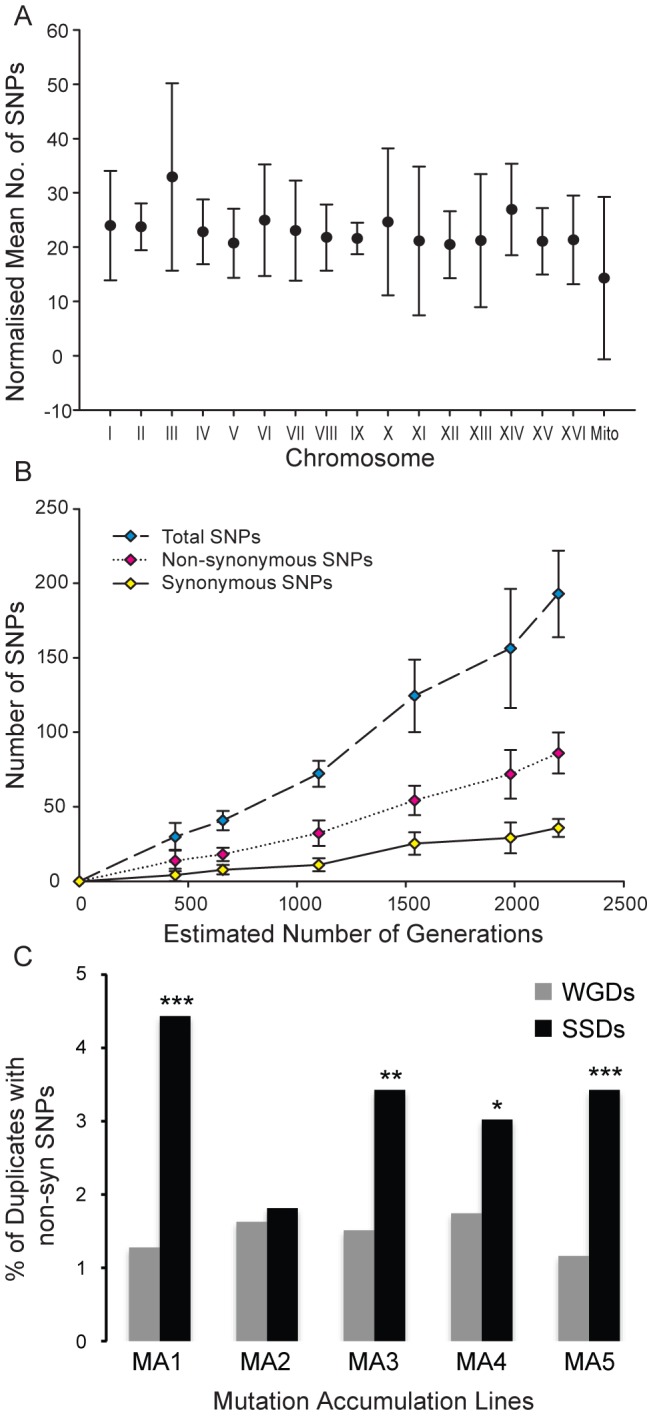
Distribution of single nucleotide polymorphisms in a mutation-accumulation experiment. (A) Normalized mean number of single nucleotide polymorphisms (SNPs) per chromosome after experimental evolution of 5 *S. cerevisiae* lineages for up to 2200 generations. The number of SNPs per chromosome was normalized by length to that of the longest chromosome (chrIV) and error bars represent the standard deviation. (B) The number of SNPs detected in the genome of each lineage increased linearly with the number of generations for total, non-synonymous and synonymous SNPs. (C) The fraction of SSDs (black columns) and WGDs (grey columns) affected by non-synonymous single nucleotide polymorphisms (Nsyn-SNPs) across the five mutation-accumulation (MA1 to MA5) experimental lines. In all five MA lines, the fraction of SSDs with Nsyn-SNPs is larger than that of WGDs. The fraction of SSDs that have fixed Nsyn-SNPs is significantly larger than that of WGDs in four of the five MA experimental lines (significance is indicated by * = *P*<0.05; ** = *P*<0.01 and *** = *P*<0.001).

To test this hypothesis in real time, we evolved for approximately 2,200 generations 5 lines of *S. cerevisiae*, all of which derived from the same ancestral strain, (100 plate-to-plate passages of single colonies). To accelerate the mutation accumulation experiment, we used an *msh2* deletion strain, which is deficient in mismatch repair (MMR) and therefore has an increased spontaneous mutation rate (see Material and Methods for details). This experiment was designed to accumulate slightly-deleterious mutations, thereby testing functional complementation between WGDs compared to that of SSDs. If non-synonymous mutations were as deleterious when they originated in WGDs as in SSDs then we should observe no significant differences in the enrichment of SSDs and WGDs for non-synonymous SNPs. We sequenced the ancestral genome and the evolved genomes at 20, 30, 50, 70, 90 and 100 passages. These lines evolved under strong bottlenecks (transferring a single colony to a fresh plate), leading to the fixation of mutations in all the yeast chromosomes (figure 6A). Synonymous and non-synonymous SNPs accumulated linearly across the evolution experiment (figure 6B), this being indicative of the effect of genetic drift on the fixation of mutations. Because of the clonal transfer nature of each line, genome-wide mutations at each isolation time (*t*) included those fixed in the previous isolation time (*t*−1).

At the end of the experiment we detected a total of 883 SNPs across the 5 lines distributed throughout the genomes (table S4) (after filtration of ancestral SNPs). Of the 883 mutations, 249 were fixed in intergenic or intronic regions, while the remaining 634 mutations affected exons. There were 158 annotated synonymous mutations in addition to 399 annotated non-synonymous mutations affecting 386 different protein-coding genes. The number of non-synonymous mutations varied between the five evolving lines, ranging between 55 (fixed in 52 protein-coding genes; approximately 0.9% of the total number of genes in the genome) and 104 (fixed in 103 protein-coding genes; approximately 1.8% of the total number of genes).

If WGDs were under stronger constraints than SSDs then deleterious non-synonymous nucleotide polymorphisms (Nsyn-SNPs) should be less likely fixed at WGD than SSDs. In addition, SSDs should fix more Nsyn-SNPs than expected because of their greater genetic robustness, as predicted by our model.

SSDs fixed more Nsyn-SNPs than WGDs in all five mutation accumulation (MA) experimental lines (Figure 6C; 21.4% of SSDs fixed Nsyn-SNPs versus 16.5% of WGDs; Fisher exact test *P* = 0.024). In four of the five MA lines, the fraction of SSDs fixing Nsyn-SNPs was significantly larger than that of WGDs (Figure 6C). On average (considering the five lines of experimental evolution), 21.4% of Non-synonymous SNPs were fixed in SSDs, against the expected value of 17.1% (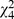 = 12.37, P<0.025). In contrast, only 16.5% of non-synonymous SNPs were fixed in WGDs against the expected value of 16.8% under the hypothesis of no functional complementation (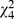 = 4.4, P>0.1). Although the protein sequence length for SSDs was slightly greater than WGDs, this was not a determining factor of whether a gene contained a Nsyn-SNP as WGDs encoded significantly larger proteins than singletons yet they accumulated similar numbers of Nsyn-SNPs. In conclusion, SSDs present greater mutational robustness than WGDs at short evolutionary time intervals, which may allow the fixation of innovative mutations despite their destabilizing effects and the rapid increase in the strength of selection constraints on these novel functions.

## Discussion

In this manuscript we have tested a new model of evolution after gene duplication. This model supports a greater likelihood of neo-functionalization after SSD than after WGD, while sub-functionalization results from the partitioning of ancestral functions which often takes place after WGD. Our model involves several predictions that allow exploring the role of the mode of duplication in the functional fates of gene copies. All these predictions were confirmed by our results, strongly supporting the model. We show that SSDs establish more interactions than WGD and than singletons, indicating their role in functional innovation because the number of such interactions in duplicates should, on average, be as large as for singletons if no novel functions have been established after duplication. we show that genes in duplicate formed by WGD share fewer genetic interactions with one another compared to gene copies from SSDs. Nevertheless, gene copies of WGDs interact with genes that are more functionally related than interaction partners of SSDs.

These results support different functional fates for WGD and SSDs. After a WGD, interacting molecules that have undergone duplication are in balance with each other and selection against deletion of one member of a pair would prevent their rapid loss [24], [64], [65]. Several pieces of evidence support dosage balance as a major force in the long-term persistence of duplicated genes. First, SSD duplicated genes are preferentially found in functional classes that complement those in which WGDs are found [8], [66], probably as a result of selection against small-scale genome duplications that upset regulatory balance. Second, large gene families seldom encode components of large complexes [67], [68]. Third retained genes in duplicate usually form part of the same protein complex [69] or metabolic pathways [70]. Finally, it has been recently shown that duplicated genes related through WGD in the human genome have rarely experienced subsequent small-scale duplications, are not prone to undergo copy number variation and are sensitive to relative quantities (dosage) [29]. This balance also imposes a constraint on the evolution of gene copies, especially those genes involved in protein complexes, because functional divergence between gene copies would immediately upset the stoichiometry of the different subunits. It is therefore more likely that each of the daughters of a WGD would specialize in a subset of the ancestral gene functions than originating novel functions, i.e, the sum of the functions for the two gene copies would perform the ancestral functions. In agreement with this, our data shows that gene copies formed by WGD are significantly less divergent than those originated by SSDs.

SSDs are more likely to innovate functions by diverging from the ancestral function. It is noteworthy that duplicates generated by SSD establish more genetic interactions than those emerging from WGD. This points to duplicates that are essential being preferentially preserved after SSD than WGD. Indeed, deletion of single genes from the set of WGD duplications had less effects on growth than SSDs, showing that WGDs play a relatively greater role in redundancy, or that WGD affects less essential genes, than SSDs [17]. However, two points remain contradictory. Firstly, if WGDs preferentially sub-functionalize, so that two gene copies now perform the job of the ancestral gene, then one would expect that both copies should be essential to perform that function. Conversely, the greater overlap between the interaction sets for SSDs should involve greater functional complementation between them, hence greater functional redundancy. One possible explanation for the contradictory patterns of genetic interactions and gene essentiality, (WGD sub-functionalize yet they are less essential), is that WGDs affect less important functions than SSDs, they are less connected in the genetic interaction networks or both of these possibilities. Our data show that, indeed WGDs do have on average fewer interactions than SSDs. Because the number of genetic interactions seem to be correlated with the sequence divergence, and sequence divergence correlates to gene essentiality, one would predict that WGDs are less essential than SSDs. The second explanation is that gene copies generated by a WGD are more functionally related, that is they functionally interact more with one another, than those emerging from SSD. This does not imply more shared interactions between both of the gene copies generated by WGD but more shared functions. Indeed, we show that WGDs share more functions than SSDs because although they share less interacting partners, these partners are nevertheless more clustered (interact between each other more) than those of duplicates generated by SSD. Greater functional interactions between WGDs has been also suggested in a previous study [25]. Also, because SSD-interaction partners are less related, SSDs perform more dispersed functions than WGDs, hinting that they may affect more cellular functions when deleted.

Our results strongly support the hypothesis that SSDs are more prone to generate novel “functions” or adaptations while WGDs are more likely to sub-functionalize by partitioning the ancestral functions between the gene daughters. This conclusion is in good agreement with recent metabolic analyses, in which SSDs were found to result in faster adaptations in anaerobic nitrogen-, phosphate- and sulphate-limited environments [70]. These authors also conclude that WGD duplicates adapt faster to new environments when the entire pathway is duplicated, thereby maintaining the required stoichiometric equilibrium. Conversely, SSDs adapt faster to new conditions when only a single gene is duplicated. That is, neo-functionalization requires less redundancy and energy through the duplication of single genes than entire pathways.

Our analyses show complex evolutionary dynamics for duplicated genes formed by SSD: Although SSDs generate novel functions, they keep significantly more ancestral subfunctions because the proportion of shared interactions between SSDs is greater than between WGDs. This also predicts greater redundancy between SSDs in terms of functional complementation. In agreement with this prediction, the experimental evolution of five unstressed lines of *S. cerevisiae* show far more fixed Nsyn-SNPs in SSDs than expected while WGDs fix as many as expected from singletons. That is, the number of possible mutations with deleterious effects is larger when fixed in WGDs than in SSDs. This further confirms that SSDs are more functionally complementary or that it is more likely to find functional complementation between SSDs than WGDs.

More functional redundancy in WGDs is difficult to reconcile with sub-functionalization, as both gene daughters are needed to perform ancestral functions. Previous observations of greater redundancy among WGDs than SSDs may be a by-product of the greater distribution of SSDs among essential genes. Essential genes are those with greater expression levels [71], [72], [73], [74], [75], [76] and number of interactions in the cell [77] and their duplication by SSDs may have a negligible effect on the stoiciometry of the cell, probably because they perform core and highly-demanding cellular functions. This would also favor the evolutionary innovation mediated by these duplications, which will impose the selective constraint necessary for their long-term survival in the genome. However, duplication of interacting partners of essential genes in a pathway may be deleterious as it would disrupt the fine-balance between these partners, many of which may be lowly-expressed, and hence WGD including these partners may be deleterious. Under this view, essential genes are more likely to preserve both gene copies after SSD, hence leading to the apparent conclusion that WGD duplicated gene copies are more redundant.

While we have shown here that evolution after gene duplication is complex, we only provide a simplistic view of how evolution proceeds in a particular timepoint and under laboratory conditions. Other parameters related to the environment and population structure may greatly influence the functional fate of duplicates. For example, a recent study comparing WGDs to SSDs in four biologically different plant species showed that the ecology of the plant may be as much of a constraint to the functional fate of duplicates as the mechanism of duplication itself [28].

Our results shed light on the role of the mode of duplication in the functional fate of duplicates and unearth the striking complexity underlying evolution by gene duplication.

### Concluding remarks

Our analyses on the distribution of functions and epistatic interactions among duplicates generated by WGD and SSD lead to the following conclusions: (1) SSDs show more complementary functions than WGDs, while being more essential than WGDs; (2) SSDs have established more epistatic interactions than singletons and WGDs, suggesting neo-functionalization after SSD; (3) WGDs have partitioned ancestral gene functions so that each gene copy performs a subset of the functions of the ancestral, pre-duplication, gene (sub-functionalization); (4) SSDs have diverged functionally more than WGDs, a fact consistent with larger functional innovations among SSDs than WGDs; (5) SSD provides more mutational robustness than WGD. We provide a mechanistic model to explain the functional fates of duplicates according to the mechanism of duplication.

## Methods

### Genetic interaction data

We used the latest update of the genetic functional chart of *S. cerevisiae* [47] (Supplementary files S4 and S5 from http://drygin.ccbr.utoronto.ca/~costanzo2009/). This functional map is based on the synthetic genetic array methodology [58], in which synthetic lethal genetic interactions are systematically mapped by producing single and double mutants [59]. In their study, Costanzo and colleagues [47] identified digenic interactions as those double mutants that show a significant deviation in fitness compared to the multiplicative fitness effects of the two single mutants, that is, epistasis (ε) [60]. Negative interactions (ε^−^) refer to those double mutants causing more severe defects than the multiplicative effects of the single mutants, with synthetic lethality being the extreme case. Positive interactions (ε^+^) are those causing less consequence than the multiplicative effects of single mutants. Defects were measured in terms of colony sizes. The updated version of the double mutants data includes more than 6×10^6^ binary genetic interactions (*GI*).

### Identifying SSD and WGDs

Paralogous pairs of duplicated genes were defined as the resulting best reciprocal hits from all-against-all BLAST-searches using BLASTP with an E-value cutoff of 1E-5 and a bit score cutoff of 50 [78]. Paralogs were further classified as ohnologs resulting from the whole genome duplication occurring in the yeast lineage 100–150 mya according to the reconciled list provided by the YGOB (Yeast Gene Order Browser, http://wolfe.gen.tcd.ie/ygob/) [79]. All other paralogs were considered to belong to SSD events.

### Measuring divergence between duplicates copies

Because SSD includes contemporaneous, older and younger duplicates than the WGD event, we corrected divergence levels of WGD and SSD normalizing them by the total divergence of the protein as follows:
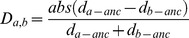
Here, divergence between the gene copies *a* and *b* is measured as the difference between the divergence of gene copy *a* and the sequence of the ancestral node (*anc*) of *a* and *b*, and that of the gene copy *b* and the ancestor, normalized by the sum of divergences. Effectively, the divergence of each gene copy to the ancestor corresponds to the length of the branch leading to that gene copy. To determine the position of the ancestor, we used as an outgroup sequence the ortholog for the duplicated gene in *Kluyveromyces polysporus* (sequence *c*). Branch length for a gene copy *a* was estimated as:
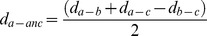



### Evolution experiments

#### Yeast strains and plasmids

The yeast strain Y06240 (BY4741; *Mata; his3D1; leu2D0; met15D0; ura3D0; msh2::kanMX4*) was obtained from Euroscarf. This *msh2* deletion strain is deficient in mismatch repair (MMR) and therefore has an increased spontaneous mutation rate. Msh2 forms a complex with Msh6 that recognizes and initiates the repair of single base mismatches or small one or two nucleotide insertions/deletions [80]. Strains lacking Msh2 are predicted to have an increased mutation rate of between 6 and 40 fold compared to wild-type [81]. Five evolving lineages of Y06240 were serially passaged onto YPD by repeated streaking, each passage resulting from re-streaking a single colony. Re-streaking was carried out every 48–72 h as required. Each lineage was passaged 100 times, which resulted in an estimated 2200 generations in total. (∼22 generations per passage for 100 passages). A glycerol stock of each lineage was prepared every 10 passages (∼220 generations) and stored at −80°C. Each evolved lineage prepared for glycerol stock was compared for growth against the starting Y06240 ancestor. Cells were grown routinely in YPD media (2% [w/v] Bacto-peptone, 1% [w/v] Yeast extract, 2% [w/v] Glucose). When solid media was required 2% [w/v] Bacto-agar was added.

#### Whole-genome sequencing

In order to map spontaneous mutations occurring during the evolution experiment, whole genome sequencing of each evolved strains was carried out. Genomes were sequenced for four strains at passages 20, 30, 50, 70, 90 and 100, and for one strain at passages 20, 50 and 90. Genomic DNA preparations were obtained using the Wizard Genomic DNA Purification Kit (Promega) as recommended by the manufacturer. DNA was quantified spectrophotometrically using a nanodrop and flurometrically using a Qubit Florometer. Illumina sequencing libraries were constructed from all strains to be sequenced by sonicating 1.5 µg of DNA using a bioruptor (Diagenode) until fragment sizes <2,000 bp were obtained. Following A-tail end-repair, Illumina adaptors were ligated to the fragment ends. Samples were run on a low melting temperature 2% agarose gel and DNA excised and eluted from the 175–225 bp range. PCR amplification was used to enrich fragments to generate final sequencing libraries at a concentration of 10 nM. An Illumina Genome Analyser II platform was used for sequencing and indexed samples were run on three separate flow cells, with two strains per lane of a flow cell using the paired-end module. After removal of the index sequence, 34 or 74 bp reads were obtained. Sequenced strains and number of quality-filtered reads obtained is available in table S4, while the chromosomal location of the SNPs is shown in table S5 (Sequences can be accessed at NCBI SRA, with accession number SRP012321).

#### Mapping mutations

Firstly, sequencing reads were converted from Illumina quality scores into Sanger quality scores. The Maq easyrun default command was used to align paired-end sequence reads to the *S. cerevisiae* genome sequence (EF2 version 59, Ensembl). The average read depth across non-gap regions for each strain is shown in table S3. The SNPs called by Maq were subsequently edited using the following criteria: a nucleotide read-depth of ≥5 was required and SNPs where the average number of hits ≠1 or where <85% of the base calls supported, were removed. This SNP calling protocol was applied for the orignal Y06240 ancestor strain and for each evolved strain to determine the total number of SNPs identified in a particular strain. SNPs that were present in the ancestral strain were removed from each evolved strain leaving the subset of SNPs that had appeared during the course of the experiment. SNPs were annotated using SNPer 1.0. This software maps the SNPs to a chromosome, gene/intergenic region, exon/intron and determines whether exonic SNPs are synonymous or non-synonymous. The number of SNPs in protein-coding genes detected at each stage of the process is listed in table S4.

## Supporting Information

Table S1 Genetic interactions and JTT-corrected amino acid divergence of duplicated genes formed by small-scale duplication (SSD) in *Saccharomyces cerevisiae*. Divergence refers to the distance from a gene copy to the most recent ancestor of the duplication event. Genes are represented by their locus tag in *S. cerevisiae*.
(XLS)

Table S2 Genetic interactions and JTT-corrected amino acid divergence of duplicated genes formed by whole genome duplication (WGD) in *Sacchromyces cerevisiae*. Divergence refers to the distance from a gene copy to the most recent ancestor of the duplication event. Genes are named according to their locus tags in *S. cerevisiae*.
(XLS)

Table S3 Sub-cellular localization of duplicated genes in *Saccharomyces cerevisiae*. Genes are named according to their locus tags in *S. cerevisiae*, with A referring to one gene copy and B to the other gene copy.
(XLS)

Table S4 Genome sequencing of five *Saccharomyces cerevisiae* experimentally evolved lines (Msh2_1 to Msh2_5). Characteristics of genomes sequences at different time points of evolution (P20, 30, 50, 70, 90 and a 100) are presented.
(XLS)

Table S5 Single Nucleotide Polymorphisms (SNPs) fixed during the experimental evolution of five lines (Msh2_1 to Msh2_5) of *Saccharomyces cerevisiae*. For each of the SNPs we show their exact chromosomal location and position.
(XLS)

## References

[1] Ohno S (1970) Evolution by Gene duplication; Olson KA, editor. Berlin: Springer-Verlag.

[2] Blanc G, Wolfe KH (2004) Functional divergence of duplicated genes formed by polyploidy during Arabidopsis evolution. Plant Cell 16: 1679–1691. 15208398 10.1105/tpc.021410 PMC514153

[3] Wendel JF (2000) Genome evolution in polyploids. Plant Mol Biol 42: 225–249. 10688139

[4] Ramsey J, Schmeske DW (1998) Pathways, mechanisms, and rates of polyploid formation in flowering plants. Annu Rev Ecol Syst 29: 35.

[5] Taylor JS, Raes J (2004) Duplication and divergence: the evolution of new genes and old ideas. Annu Rev Genet 38: 615–643. 15568988 10.1146/annurev.genet.38.072902.092831

[6] Lespinet O, Wolf YI, Koonin EV, Aravind L (2002) The role of lineage-specific gene family expansion in the evolution of eukaryotes. Genome Res 12: 1048–1059. 12097341 10.1101/gr.174302 PMC186617

[7] Holub EB (2001) The arms race is ancient history in Arabidopsis, the wildflower. Nat Rev Genet 2: 516–527. 11433358 10.1038/35080508

[8] Maere S, De Bodt S, Raes J, Casneuf T, Van Montagu M, et al (2005) Modeling gene and genome duplications in eukaryotes. Proc Natl Acad Sci U S A 102: 5454–5459. 15800040 10.1073/pnas.0501102102 PMC556253

[9] Aoki S, Uehara K, Imafuku M, Hasebe M, Ito M (2004) Phylogeny and divergence of basal angiosperms inferred from APETALA3- and PISTILLATA-like MADS-box genes. J Plant Res 117: 229–244. 15138844 10.1007/s10265-004-0153-7

[10] Kim S, Yoo MJ, Albert VA, Farris JS, Soltis PS, et al (2004) Phylogeny and diversification of B-function MADS-box genes in angiosperms: evolutionary and functional implications of a 260-million-year-old duplication. Am J Bot 91: 2102–2118. 21652358 10.3732/ajb.91.12.2102

[11] Kramer EM, Dorit RL, Irish VF (1998) Molecular evolution of genes controlling petal and stamen development: duplication and divergence within the APETALA3 and PISTILLATA MADS-box gene lineages. Genetics 149: 765–783. 9611190 10.1093/genetics/149.2.765 PMC1460198

[12] Purugganan MD, Rounsley SD, Schmidt RJ, Yanofsky MF (1995) Molecular evolution of flower development: diversification of the plant MADS-box regulatory gene family. Genetics 140: 345–356. 7635298 10.1093/genetics/140.1.345 PMC1206560

[13] Hoegg S, Brinkmann H, Taylor JS, Meyer A (2004) Phylogenetic timing of the fish-specific genome duplication correlates with the diversification of teleost fish. J Mol Evol 59: 190–203. 15486693 10.1007/s00239-004-2613-z

[14] Otto SP, Whitton J (2000) Polyploid incidence and evolution. Annu Rev Genet 34: 401–437. 11092833 10.1146/annurev.genet.34.1.401

[15] Moore RC, Grant SR, Purugganan MD (2005) Molecular population genetics of redundant floral-regulatory genes in Arabidopsis thaliana. Mol Biol Evol 22: 91–103. 15371526 10.1093/molbev/msh261

[16] Zhang J (2003) Evolution by gene duplication: an update. Trends in Ecology and Evolution 18: 292–298.

[17] Gu Z, Steinmetz LM, Gu X, Scharfe C, Davis RW, et al (2003) Role of duplicate genes in genetic robustness against null mutations. Nature 421: 63–66. 12511954 10.1038/nature01198

[18] Conant GC, Wolfe KH (2008) Turning a hobby into a job: how duplicated genes find new functions. Nat Rev Genet 9: 938–950. 19015656 10.1038/nrg2482

[19] Force A, Lynch M, Pickett FB, Amores A, Yan YL, et al (1999) Preservation of duplicate genes by complementary, degenerative mutations. Genetics 151: 1531–1545. 10101175 10.1093/genetics/151.4.1531 PMC1460548

[20] Barkman T, Zhang J (2009) Evidence for escape from adaptive conflict? Nature 462: E1 discussion E2–3. 20010636 10.1038/nature08663

[21] Des Marais DL, Rausher MD (2008) Escape from adaptive conflict after duplication in an anthocyanin pathway gene. Nature 454: 762–765. 18594508 10.1038/nature07092

[22] He X, Zhang J (2005) Rapid subfunctionalization accompanied by prolonged and substantial neofunctionalization in duplicate gene evolution. Genetics 169: 1157–1164. 15654095 10.1534/genetics.104.037051 PMC1449125

[23] Francino MP (2005) An adaptive radiation model for the origin of new gene functions. Nat Genet 37: 573–577. 15920518 10.1038/ng1579

[24] Freeling M, Thomas BC (2006) Gene-balanced duplications, like tetraploidy, provide predictable drive to increase morphological complexity. Genome Res 16: 805–814. 16818725 10.1101/gr.3681406

[25] Hakes L, Pinney JW, Lovell SC, Oliver SG, Robertson DL (2007) All duplicates are not equal: the difference between small-scale and genome duplication. Genome Biol 8: R209. 17916239 10.1186/gb-2007-8-10-r209 PMC2246283

[26] Innan H, Kondrashov F (2010) The evolution of gene duplications: classifying and distinguishing between models. Nat Rev Genet 11: 97–108. 20051986 10.1038/nrg2689

[27] Lynch M, O'Hely M, Walsh B, Force A (2001) The probability of preservation of a newly arisen gene duplicate. Genetics 159: 1789–1804. 11779815 10.1093/genetics/159.4.1789 PMC1461922

[28] Carretero-Paulet L, Fares MA (2012) Evolutionary dynamics and functional specialization of plant paralogs formed by whole and small-scale genome duplications. Mol Biol Evol 10.1093/molbev/mss162 22734049

[29] Makino T, McLysaght A (2010) Ohnologs in the human genome are dosage balanced and frequently associated with disease. Proc Natl Acad Sci U S A 107: 9270–9274. 20439718 10.1073/pnas.0914697107 PMC2889102

[30] Lynch M, Conery JS (2000) The evolutionary fate and consequences of duplicate genes. Science 290: 1151–1155. 11073452 10.1126/science.290.5494.1151

[31] Gu X (1999) Statistical methods for testing functional divergence after gene duplication. Mol Biol Evol 16: 1664–1674. 10605109 10.1093/oxfordjournals.molbev.a026080

[32] Gu X (2001) Mathematical modeling for functional divergence after gene duplication. J Comput Biol 8: 221–234. 11535174 10.1089/10665270152530827

[33] Kellis M, Birren BW, Lander ES (2004) Proof and evolutionary analysis of ancient genome duplication in the yeast Saccharomyces cerevisiae. Nature 428: 617–624. 15004568 10.1038/nature02424

[34] Costanzo M, Baryshnikova A, Myers CL, Andrews B, Boone C (2011) Charting the genetic interaction map of a cell. Curr Opin Biotechnol 22: 66–74. 21111604 10.1016/j.copbio.2010.11.001

[35] Giot L, Bader JS, Brouwer C, Chaudhuri A, Kuang B, et al (2003) A protein interaction map of Drosophila melanogaster. Science 302: 1727–1736. 14605208 10.1126/science.1090289

[36] Ito T, Chiba T, Ozawa R, Yoshida M, Hattori M, et al (2001) A comprehensive two-hybrid analysis to explore the yeast protein interactome. Proc Natl Acad Sci U S A 98: 4569–4574. 11283351 10.1073/pnas.061034498 PMC31875

[37] Ito T, Chiba T, Yoshida M (2001) Exploring the protein interactome using comprehensive two-hybrid projects. Trends Biotechnol 19: S23–27. 11780966 10.1016/S0167-7799(01)01790-5

[38] Ito T, Ota K, Kubota H, Yamaguchi Y, Chiba T, et al (2002) Roles for the two-hybrid system in exploration of the yeast protein interactome. Mol Cell Proteomics 1: 561–566. 12376571 10.1074/mcp.r200005-mcp200

[39] Uetz P, Giot L, Cagney G, Mansfield TA, Judson RS, et al (2000) A comprehensive analysis of protein-protein interactions in Saccharomyces cerevisiae. Nature 403: 623–627. 10688190 10.1038/35001009

[40] Gavin AC, Aloy P, Grandi P, Krause R, Boesche M, et al (2006) Proteome survey reveals modularity of the yeast cell machinery. Nature 440: 631–636. 16429126 10.1038/nature04532

[41] Gavin AC, Bosche M, Krause R, Grandi P, Marzioch M, et al (2002) Functional organization of the yeast proteome by systematic analysis of protein complexes. Nature 415: 141–147. 11805826 10.1038/415141a

[42] Ho Y, Gruhler A, Heilbut A, Bader GD, Moore L, et al (2002) Systematic identification of protein complexes in Saccharomyces cerevisiae by mass spectrometry. Nature 415: 180–183. 11805837 10.1038/415180a

[43] Krogan NJ, Peng WT, Cagney G, Robinson MD, Haw R, et al (2004) High-definition macromolecular composition of yeast RNA-processing complexes. Mol Cell 13: 225–239. 14759368 10.1016/s1097-2765(04)00003-6

[44] Wagner A (2002) Asymmetric functional divergence of duplicate genes in yeast. Mol Biol Evol 19: 1760–1768. 12270902 10.1093/oxfordjournals.molbev.a003998

[45] Conant GC, Wolfe KH (2006) Functional partitioning of yeast co-expression networks after genome duplication. PLoS Biol 4: e109 doi:10.1371/journal.pbio.0040109. 16555924 10.1371/journal.pbio.0040109 PMC1420641

[46] Guan Y, Dunham MJ, Troyanskaya OG (2007) Functional analysis of gene duplications in Saccharomyces cerevisiae. Genetics 175: 933–943. 17151249 10.1534/genetics.106.064329 PMC1800624

[47] Costanzo M, Baryshnikova A, Bellay J, Kim Y, Spear ED, et al (2010) The genetic landscape of a cell. Science 327: 425–431. 20093466 10.1126/science.1180823 PMC5600254

[48] Dixon SJ, Costanzo M, Baryshnikova A, Andrews B, Boone C (2009) Systematic mapping of genetic interaction networks. Annu Rev Genet 43: 601–625. 19712041 10.1146/annurev.genet.39.073003.114751

[49] Dobzhansky T (1946) Genetics of natural populations. XIII. Recombination and variability in populations of Drosophila pseudoobscura. Genetics 31: 1. 20985721 10.1093/genetics/31.3.269 PMC1209328

[50] Novick P, Osmond BC, Botstein D (1989) Suppressors of yeast actin mutations. Genetics 121: 659–674. 2656401 10.1093/genetics/121.4.659 PMC1203651

[51] VanderSluis B, Bellay J, Musso G, Costanzo M, Papp B, et al (2010) Genetic interactions reveal the evolutionary trajectories of duplicate genes. Mol Syst Biol 6: 429. 21081923 10.1038/msb.2010.82 PMC3010121

[52] Jiang H, Xu L, Gu Z (2011) Growth of novel epistatic interactions by gene duplication. Genome Biol Evol 3: 295–301. 21402864 10.1093/gbe/evr016 PMC3274824

[53] Gu X (2003) Evolution of duplicate genes versus genetic robustness against null mutations. Trends Genet 19: 354–356. 12850437 10.1016/S0168-9525(03)00139-2

[54] Ihmels J, Collins SR, Schuldiner M, Krogan NJ, Weissman JS (2007) Backup without redundancy: genetic interactions reveal the cost of duplicate gene loss. Mol Syst Biol 3: 86. 17389874 10.1038/msb4100127 PMC1847942

[55] DeLuna A, Vetsigian K, Shoresh N, Hegreness M, Colon-Gonzalez M, et al (2008) Exposing the fitness contribution of duplicated genes. Nat Genet 40: 676–681. 18408719 10.1038/ng.123

[56] Dean EJ, Davis JC, Davis RW, Petrov DA (2008) Pervasive and persistent redundancy among duplicated genes in yeast. PLoS Genet 4: e1000113 doi:10.1371/journal.pgen.1000113. 18604285 10.1371/journal.pgen.1000113 PMC2440806

[57] Musso G, Costanzo M, Huangfu M, Smith AM, Paw J, et al (2008) The extensive and condition-dependent nature of epistasis among whole-genome duplicates in yeast. Genome Res 18: 1092–1099. 18463300 10.1101/gr.076174.108 PMC2493398

[58] Tong AH, Evangelista M, Parsons AB, Xu H, Bader GD, et al (2001) Systematic genetic analysis with ordered arrays of yeast deletion mutants. Science 294: 2364–2368. 11743205 10.1126/science.1065810

[59] Tong AH, Lesage G, Bader GD, Ding H, Xu H, et al (2004) Global mapping of the yeast genetic interaction network. Science 303: 808–813. 14764870 10.1126/science.1091317

[60] Mani R, St Onge RP, Hartman JLt, Giaever G, Roth FP (2008) Defining genetic interaction. Proc Natl Acad Sci U S A 105: 3461–3466. 18305163 10.1073/pnas.0712255105 PMC2265146

[61] Wolfe KH, Shields DC (1997) Molecular evidence for an ancient duplication of the entire yeast genome. Nature 387: 708–713. 9192896 10.1038/42711

[62] Gu X (2001) Maximum-likelihood approach for gene family evolution under functional divergence. Mol Biol Evol 18: 453–464. 11264396 10.1093/oxfordjournals.molbev.a003824

[63] Guldener U, Munsterkotter M, Kastenmuller G, Strack N, van Helden J, et al (2005) CYGD: the Comprehensive Yeast Genome Database. Nucleic Acids Res 33: D364–368. 15608217 10.1093/nar/gki053 PMC540007

[64] Birchler JA, Riddle NC, Auger DL, Veitia RA (2005) Dosage balance in gene regulation: biological implications. Trends Genet 21: 219–226. 15797617 10.1016/j.tig.2005.02.010

[65] Birchler JA, Veitia RA (2007) The gene balance hypothesis: from classical genetics to modern genomics. Plant Cell 19: 395–402. 17293565 10.1105/tpc.106.049338 PMC1867330

[66] Davis JC, Petrov DA (2004) Preferential duplication of conserved proteins in eukaryotic genomes. PLoS Biol 2: e55 doi:10.1371/journal.pbio.0020055. 15024414 10.1371/journal.pbio.0020055 PMC368158

[67] Papp B, Pal C, Hurst LD (2003) Dosage sensitivity and the evolution of gene families in yeast. Nature 424: 194–197. 12853957 10.1038/nature01771

[68] Yang J, Lusk R, Li WH (2003) Organismal complexity, protein complexity, and gene duplicability. Proc Natl Acad Sci U S A 100: 15661–15665. 14660792 10.1073/pnas.2536672100 PMC307624

[69] Aury JM, Jaillon O, Duret L, Noel B, Jubin C, et al (2006) Global trends of whole-genome duplications revealed by the ciliate Paramecium tetraurelia. Nature 444: 171–178. 17086204 10.1038/nature05230

[70] van Hoek MJ, Hogeweg P (2009) Metabolic adaptation after whole genome duplication. Mol Biol Evol 26: 2441–2453. 19625390 10.1093/molbev/msp160

[71] Drummond DA, Bloom JD, Adami C, Wilke CO, Arnold FH (2005) Why highly expressed proteins evolve slowly. Proc Natl Acad Sci U S A 102: 14338–14343. 16176987 10.1073/pnas.0504070102 PMC1242296

[72] Drummond DA, Raval A, Wilke CO (2006) A single determinant dominates the rate of yeast protein evolution. Mol Biol Evol 23: 327–337. 16237209 10.1093/molbev/msj038

[73] Krylov DM, Wolf YI, Rogozin IB, Koonin EV (2003) Gene loss, protein sequence divergence, gene dispensability, expression level, and interactivity are correlated in eukaryotic evolution. Genome Res 13: 2229–2235. 14525925 10.1101/gr.1589103 PMC403683

[74] Lemos B, Bettencourt BR, Meiklejohn CD, Hartl DL (2005) Evolution of proteins and gene expression levels are coupled in Drosophila and are independently associated with mRNA abundance, protein length, and number of protein-protein interactions. Mol Biol Evol 22: 1345–1354. 15746013 10.1093/molbev/msi122

[75] Pal C, Papp B, Hurst LD (2001) Highly expressed genes in yeast evolve slowly. Genetics 158: 927–931. 11430355 10.1093/genetics/158.2.927 PMC1461684

[76] Drummond DA, Wilke CO (2008) Mistranslation-induced protein misfolding as a dominant constraint on coding-sequence evolution. Cell 134: 341–352. 18662548 10.1016/j.cell.2008.05.042 PMC2696314

[77] Hwang YC, Lin CC, Chang JY, Mori H, Juan HF, et al (2009) Predicting essential genes based on network and sequence analysis. Mol Biosyst 5: 1672–1678. 19452048 10.1039/B900611G

[78] Altschul SF, Madden TL, Schaffer AA, Zhang J, Zhang Z, et al (1997) Gapped BLAST and PSI-BLAST: a new generation of protein database search programs. Nucleic Acids Res 25: 3389–3402. 9254694 10.1093/nar/25.17.3389 PMC146917

[79] Byrne KP, Wolfe KH (2005) The Yeast Gene Order Browser: combining curated homology and syntenic context reveals gene fate in polyploid species. Genome Res 15: 1456–1461. 16169922 10.1101/gr.3672305 PMC1240090

[80] Habraken Y, Sung P, Prakash L, Prakash S (1996) Binding of insertion/deletion DNA mismatches by the heterodimer of yeast mismatch repair proteins MSH2 and MSH3. Curr Biol 6: 1185–1187. 8805366 10.1016/s0960-9822(02)70686-6

[81] Kunz BA, Ramachandran K, Vonarx EJ (1998) DNA sequence analysis of spontaneous mutagenesis in Saccharomyces cerevisiae. Genetics 148: 1491–1505. 9560369 10.1093/genetics/148.4.1491 PMC1460101

